# The Use of Clinical Scores in the Management of Immune Thrombocytopenic Purpura in Children

**DOI:** 10.3389/fped.2022.870064

**Published:** 2022-05-09

**Authors:** Vasile Eduard Roşu, Elena-Lia Spoială, Tamara Solange Roşu, Anca-Viorica Ivanov, Adriana Mocanu, Alecsandra Munteanu, Vasile Valeriu Lupu, Ingrith Miron, Cristina Gavrilovici

**Affiliations:** ^1^Department of Mother and Child, “Grigore. T. Popa” University of Medicine and Pharmacy of Iaşi, Iaşi, Romania; ^2^Sf.Maria Emergency Hospital for Children, Iaşi, Romania; ^3^Department of Nursing, “Grigore. T. Popa"” University of Medicine and Pharmacy of Iaşi, Iaşi, Romania

**Keywords:** immune thrombocytopenic purpura, children, bleeding score, predictive score, platelet, risk factors, diagnostics

## Abstract

While the majority of children with recently diagnosed ITP have a benign, self-limiting condition, most often with a spontaneously recovery, 40% of children with ITP progress toward persistent ITP and 10–20% goes toward chronicity. Several clinical scores have been developed with the aim to perform a better monitoring outcome or to differentiate transient vs. persistent ITP (e.g., Donato score). Our paper aims to describe and to compare the most important scores used in the management of ITP in children: bleeding severity scores and chronicity prediction scores. These scores include a combination of different already known risk factors: age, gender, presence of a previous infections or vaccination, bleeding grade, type of onset, platelet count at diagnosis. The real utility of these scores has been a matter of debate and no consensus has been reached so far as to their necessity to be implemented as compulsory tool in the care of children with ITP.

## Introduction

Immune thrombocytopenia in children (ITP) is an acute autoimmune bleeding disease characterized by decreased platelet counts <100 × 10^9^/L (1). The incidence of ITP in childhood varies from 1.6 to 6 per 100,000 children (2, 3). Most children with recently diagnosed ITP have a benign, self-limiting condition which usually recovers spontaneously, with minimal morbidity and mortality (4, 5). The frequency of severe hemorrhage episodes in childhood is ~1/800 (1), the intracranial hemorrhage being the most important, thought rare complication (4, 5), occurring in both newly diagnosed and in persistent/chronic ITP patients (6); 40% of children with ITP progress toward persistent ITP exhibiting a low platelet count and hemorrhage episodes beyond 3 months, and 10–20% goes toward chronicity.

While investigations into immune functions in ITP (7, 8), aiming for a better prediction of platelet count responses have been exploited and included in nowadays ITP management (9–11), a lack of consensus among clinical scores and use in ITP monitoring still exists (10). Several clinical scores have been developed including a combination of different already known risk factors: age, gender, presence of a previous infections or vaccination, bleeding grade, type of onset, platelet count at diagnosis (1, 12–16). These scores aim either to assess bleeding severity, to perform a better monitoring or to predict transient vs. persistent ITP (1, 12–14). More precisely the bleeding assessment scores could help in distinguish between children who can be monitored with watchful waiting and the ones needing pharmacological treatment. The other type of scores are the predictive scores useful for identifying patients that present high risk of developing persistent and chronic ITP.

However, the usefulness of the clinical score has been a matter of debate (12–14) and no consensus has been reached so far as to their necessity to be implemented as a compulsory tool in the care of children with ITP. Thus, their (in) utility needs to be proved. This literature review aims to describe and to compare the most important clinical scores in ITP in children highlighting the advantages and disadvantages of their application in clinical setting.

## Materials and Methods

We performed a literature review on PubMed from the time of their initiation to November 2021, to capture the original research studies investigating the clinical scores in immune thrombocytopenic purpura in children. We used the following search terms (“immune thrombocytopenia”) AND [(“clinical score”) OR (“score”)] AND [(“children”) OR (“pediatric”) OR (“pediatric”)]. All the articles in English and French language were included.

## Results

The literature search retrieved 97 references (see Supplementary File 1). All the articles have been screened by title and abstracts. We removed studies performed on animals, case reports and articles about ITP in adults. After reading 42 full articles, 27 were excluded due to lack of reference to clinical scores or other limitations (results limited to quality of life or treatment options with no relevant approach on clinical scores). We also added 6 studies after analyzing the references. In total, 21 papers were included in this review.

## Discussion

In order to analyze the available clinical score for ITP in children, we have classified them in two categories: bleeding severity assessment scores and predictive clinical scores.

### Bleeding Severity Assessment Scores

In addition to platelet normalization, it is important to consider in the management decision the bleeding assesment assessment in the affected child (Table 1—See Supplementary File 2).

#### The Buchanan Bleeding Scores

As it was commonly agreed that bleeding isn't necessarily related to a low platelet count, Buchanan and Adix (17) tried to elaborate a semi-quantitative assessment instrument for the bleeding symptoms in ITP without relation to platelet count. Their bleeding scale provides grades (from 0 to 5) of cutaneous and mucosal bleeding (oral and epistaxis). Grade 1 refers to minor skin bleeding, minor petechiae on palate or buccal mucosa and no active mucosal bleeding. Grade 2 includes the possibility of small bruises (<3 cm diameter) buccal haemorrhagic bullae or infiltrates, mild active bleeding. Grade 3 involves overt mucosal bleeding (including menorrhagia or gastrointestinal bleeding). Grade 4 includes severe mucosal bleeding or internal hemorrhage, requiring intensive care and grade 5 refers to life—threatening hemorrhage at any site (17).

According to the guidelines of the American Society of Hematology for ITP treatment, Buchanan bleeding score of 0–2 requires only supervision, patients with a score of 4 or 5 need pharmacological treatment, while the therapeutic decision for those with a score of 3 remains controversial.

Schoettler et al. (4) designed a standardized clinical assessment and management plan (SCAMP) using a modified version of Buchanan score, in an attempt to increase the percentage of patients who only need monitoring, to reduce practice variation, to identify nonconformities in clinical decision making, and decrease economic costs by increasing the observation rates in low-risk cases. In this modified score, the grade 3 bleeding was divided into high and low-risk subgroups. High risk grade 3 comprise epistaxis lasting more than 5 min, haematochezia, haematuria, significant menorrhagia, painful oral purpura (4).

The eligibility criteria in this study were: patients aged 1–16 years with a clinical diagnosis of immune thrombocytopenia, with platelet count below 30 × 10^9^/L, duration of thrombocytopenia under 4 weeks and no treatment until then. Two standardized plans for the assessment and management of autoimmune thrombocytopenia were implemented for these eligible patients: the first one (applied in a sample of 40 patients with the above criteria) used the classic Buchanan score; the second one used the revised Buchanan score in a sample of 31 patients. The observation decision rate (as a therapeutic option) increased from 40 to 74%.

Thus, the modified Buchanan score (with grade 3 split into moderate-low and moderate-high) could lead to increased observation rates in patients newly diagnosed with immune thrombocytopenia that would require only monitoring, therefore having the potential to lowert the quality of life.

This study confirmed that the score developed by Buchanan and Addix is usefull in clinical decision making but the results are to be verified in more extensive randomized trials.

#### Page et al.'s ITP Bleeding Score (IBLS) Score

Page et al. (18) aimed to implement a bleeding scale and correlate it with the bleeding severity and platelets variable (count and size) in a pilot study of 65 patients, including both children and adults. The bleeding scale comprises grades of severity (from 0 to 2) assessed in 9 anatomical sites: skin, oral (each of them recorded from history and physical exam), epistaxis, pulmonary, gastro-intestinal, gynecological, intracranial hemorrhage and subconjuctival hemorrhage. were assessed. Oral bleeding was more common in patients who had more cutaneous bleeding. For the 5/6 sites, platelet count and large platelet count correlated with the bleeding grade. However, when visits with platelet counts <30 × 10^9^/L were independently assessed, the platelet count and large size platelet count were found to be unrelated with bleeding events. Thus, by considering 11 bleeding sites, this score creates a larger picture of bleeding symptoms than Buchanan scale (which consider only skin and mucosal bleeding).

#### The International Working Group (IWG) on Immune Thrombocytopenia Bleeding Assessment Tool

The IWG acknowledges that for evaluating ITP treatment efficiency, the platelet count threshold is not adequate if used as a single parameter for making such decisions (19). Historically speaking, the World Health Organization (WHO) bleeding scale (20) (and its many variations) was used in patients with thrombocytopenia secondary to chemotherapy, but this has limited sensitivity when applied to ITP patients. As a result, IWG created an ITP-specific Bleeding Assessment Tool (ITP-BAT), version 1.0, based on the bleeding location and severity grading, with aim of performing a better evaluation of treatment effectiveness. The hemorrhagic manifestations are classified into three groups: skin (S), mucosal (M), and organ (O). The scale varies from 0 to 4 for epistaxis and for organ bleeding, except ocular and intracranial bleeding (grade 0 and 2 to 4). The remaining bleeding sites (cutaneous and mucosal) were classified in four grades (0–3). Grade 5 is related to any fatal bleeding The SMO Grade (SMOG) index is calculated by choosing the highest grade for each location. The IWG considered bleeding manifestation as being “severe or clinically relevant” if it is grade 3 for skin and/or grade 2 or higher for mucosal domains and/or higher than grade 1 for organ domain (S.2 and/or M.1 and/or O.1). Overall the merit of the ITP- BAT derives first from the harmonization of terminology and a detailed definition of bleeding in ITP. For instance they recommends against the use of terminologies such as “wet” or “dry” purpura due to lack in precision. Instead they propose grading the different types of skin, mucosal and organ bleeding.

Before the widespread application in clinical practice, the proposed BAT will need to be validated in well conducted, prospective clinical investigations.

### Predictive Clinical Scores

The need to find ways of predicting clinical course of ITP is stated by an important number of publications on the subject. While researchers work to find molecular markers linked to predisposition for chronicity of immune thrombocytopenia a few clinical scores have been developed with the same aim. The results of different studies found several clinical items relevant for elaborating predictive scores for evolution of patients with ITP (Table 2—See Supplementary File 3).

These scores are useful for differentiating patients that could be monitored only by watchful waiting from those who need treatment and also to help lifting the burden of fear and anxiety amongst patients and their families in relation with the clinical course of the disease.

#### Edslev et al. Score: The Nordic Idiopathic Thrombocytopenic Purpura Study (NOPHO Group)

The NOPHO study (2007) (21) involved 98 pediatric centers form five north European countries (Norway, Iceland, Finland, Denmark and Sweden) over 2 years, with 506 newly diagnosed children with ITP, aiming to predict brief, uneventful courses based on six clinical features: 1. male gender (weight 1); 2. wet purpura (weight 1); 3. platelet count at diagnosis <5 x 10^9^/L (weight 2); 4. post-infection (weight 2); 5. age <10 years (weight 3); 6. abrupt onset (symptoms for <14 day, weight 5). The overall score varied from 0 to 14: low (0–4), intermediate (5–9), and high (10–14). A short duration of thrombocytopenia was defined as <3 months, while persistent duration of thrombocytopenia, more than 3 months. The cases with high scores (10–14) had a 73% chance of having a short disease duration, whereas those with intermediate values (5–9) had a 53 % chance, and those with low scores (0–4) had a 19% chance. All of these were linked to a duration <3 months. The findings revealed that the higher the score, the higher probability of short course of the thrombocytopenia. Thus, children with high scores, whose periods of severe thrombocytopenia are unlikely to remain longer than 1 month, may be considered for observation, while cases with low scores may be prone for a longer course of treatment (21). However, for the results of these study the statistical interval of confidence is not provided, therefore is difficult to state that these data of correlation higher scores with short duration of the disease are reliable or may need further validation.

While chronic ITP is defined as thrombocytopenia that lasts longer than 6 months, “The Intercontinental Study Group” recommended that the cut-off point be adjusted to 1 year due to the important percentage of recovery between 6 and 12 months (22). Yet, results from a previous Nordic study, on the other hand, suggested that a 3-month cut-off is preferable, dividing the infants into those who have a brief and usually a silent course and those who have prolonged thrombocytopenia with resulting morbidity (23). Due to this lack of consensus, Edslev et al. (21) recognize the importance of a reliable short-term prognosis at the time of diagnosis. Nevertheless, the score developed by NOPHO group may not be applicable to cases with milder degrees of thrombocytopenia.

#### Donato et al.'s Score

A slightly modified version of NOPHO score was used by Donato et al. (19) in a large study over a sample of 1,683 patients with ITP treated in 12 centers in Argentina aiming to assess its predictive values. The threshold for considering a low platelet count <10 × 10^9^/L instead of <5 × 10^9^/L was the only difference between the two clinical tools. The authors aimed to analyse how beneficial this score was in their population for predicting ITP duration <6 months in 3 age groups: <12 months, 1–8 years, and over 9 years old. The choice of 6 months as predictive threshold was based on previous studies that found a high percentage of spontaneous remission after 6 months from diagnosis (5, 9, 13, 21, 24).

In their cohort, high scores had a predictive value of 84% for acute ITP, results that are similar to those reported by the NOPHO Group. This score seemed to be beneficial in predicting acute ITP in children aged more than 1-year-old, but no predictive value was established in infants <12 months.

Chronic ITP was detected in 28.5% cases. 79 children underwent a splenectomy; 55 of them (69.5%) had normal platelet counts. 107 of remaining 325 non-splenectomised children (32.9%) experienced spontaneous remission between 6 months and 11 years after diagnosis; 48 of them (44.9%) recovered between 6th and 12th months from diagnosis. The recovery rate in this cohort was 82.9%, including short-term and long-term spontaneous and splenectomy-induced remissions. Considering that only 20% of children do not obtain spontaneous remission, it was recommended that the indication for splenectomy should be carefully considered, given that the majority of patients will most likely achieve remission within a few years. In Donato's study splenectomy was not recommended before 12 months after diagnosis, excepting the life-threatening bleeding situations. The same precautions were also applicable to other therapy choices, such as cyclosporine A, rituximab, or azathioprine.

Since many children with chronic ITP achieved spontaneous long-term remission, mainly within 6–12 months from diagnosis, it was suggested that the cut-off value to define chronic ITP should be 12 months from diagnosis (23).

#### Revel-Vilk et al.'s Score

Revel-Vilk et al. (20) conducted a study on 472 children with the scope of developing a more simplified score (compared with the NOPHO group) and to compare it with the Nordic score for resolution prediction at 3, 6, and 12 months after the initial presentation. The score included just the age and the duration of haemorrhagic manifestations at the moment of diagnosis. The following parameters were found to be the most important predictors of ITP resolution at 3, 6, and 12 months: children aged <10 years old and short onset (<2 weeks of bleeding). Children under the age of 10 years who presented with abrupt onset of bleeding symptoms (<2 weeks) were classified as “low risk”. Children under the age of ten who presented with non-abrupt onset (bleeding lasting more than 2 weeks) or children over the age of ten with abrupt onset were considered as intermediate risk. High-risk cases were those over the age of ten who presented with non-abrupt onset. The most important predictors for ITP resolution at all 3 moments were age <10 years at onset and sudden onset. The risk to develop chronic ITP was significantly higher for children younger than 10 years' old who had non-abrupt onset (>2 weeks bleeding symptoms) compared with those with abrupt onset.

Although not included in the proposed simplified score, the level of thrombocytopenia at diagnosis was found to be predictive for early resolution (3 and 6 months from diagnosis) but not for chronic ITP. Children with thrombocytopenia <10 × 10^9^/L had 70% probability of ITP resolution at 3 months from diagnosis compared with a significantly lower probability, 55%, for children with higher platelet count at diagnosis which demonstrates that platelet count at presentation was a significant predictor for early resolution (3 and 6 months from diagnosis) but not for development of chronic ITP.

The current simplified score had a similar predictive power for early ITP resolution with the Nordic score. However, the simplified prediction method was considerably more sensitive than the Nordic score for predicting ITP resolution at 1 year without loss of specificity in this population (19, 23).

The new simplified score was also tested in relation with the initial management of the disease. Observation, corticosteroids, intravenous anti-D globulin IGIV were among the recommended treatments. ITP resolution was defined as platelet count over 100 × 10^9^/L, without any relationship with recent treatment (i.e., at least 2 months after last therapy). Children who initially were observed, without therapy, had higher platelet counts and lower rate of mucosal bleeding at diagnosis. Resolution of ITP at 3, 6, and 12 months from diagnosis, adjusted for age, duration of symptoms, and initial platelet count was not different between children who were initially treated with IGIV compared with those treated initially with corticosteroid, anti-D globulin intravenous, or observation. The need for second type of therapy was similar in children treated initially with IGIV, anti-D globulin intravenous, and corticosteroids (19).

#### The Childhood ITP Recovery Score

Schmidt et al. (25) created a prognostic tool aiming to predict transient vs. persistent ITP in childhood. The results from the NOPHO (Nordic Pediatric Hematology—Oncology) ITP study were used to develop the score, which was then prospectively validated in the TIKI cohort (Treatment With or Without Intravenous Immunoglobulins in Kids).

The following predictors were included: age, gender, history of previous infection, history of preceding vaccination, insidious disease onset, platelet count at the diagnosis, mucosal bleeding (presence of “wet purpura” or a modified Buchanan score ≥ 3). The accuracy of this score stems from the fact that age and platelet count have been used as continuous variables (and not dichotomic ones, as in previous scores), and this tool has been tested prospectively.

By introducing these parameters, the tool calculates the probability to develop transient ITP. The strongest predictor of transient ITP of all the studied variables was the absence of an insidious disease onset. The group with the lowest Childhood ITP Recovery Score was clearly associated with a lower chance for complete recovery during follow-up, and included a high proportion of patients with chronic ITP at 1 year after diagnosis. The Childhood ITP Recovery Score may be applied using an online calculator (http://www.itprecoveryscore.org).

## Conclusions

ITP is a disease that may deeply affect the child and family quality of life. Many attempts have been made to characterize the bleeding severity as well as the prediction of chronicity. No unique clinical tool or score have been proven an absolute power in ITP management in children.

The initial scores considered only skin and mucosal bleeding with no relation to platelet count (Buchanan score), and later on, more complex scores have been created and validated, adding other clinical features.

The scores assessing the bleeding severity may be useful in deciding the approach of the patient: observation or pharmacological treatment, while predictive scores have more implication on advising the families and preparing them for the eventuality of dealing with a chronic disease. It is probably best that patients that seem to have an evolution toward chronicity are better followed by hematologist more experienced from specialized centers.

The different studies used similar parameters to develop predictive scores. Thus, the Nordic score (NOPHO) included age <10 years old, male gender, platelet count <5 × 10^9^/L, history of infection, abrupt onset, wet purpura and demonstrated prediction for short duration of ITP. Modified versions of this score have been used by Donato et al. (19), Schmidt et al., (25) (Childhood ITP recovery score) and Revel-Vilk et al. (20). Donato modified the platelet threshold from <5 × 10^9^/L from Nordic score, into <10 × 10^9^/L and performed separate prediction in three age groups, thus demonstrating a more accurate prediction of development of acute ITP in patients older than 1 year. Revel-Vilk et al. (20) produced a simplified version of Nordic score, using only age and duration of bleeding (abrupt onset) as predictors, demonstrating an increased insensitivity for prediction of ITP 1 year after diagnoses than Nordic score. Their conclusion was that the use of just 2 of 6 criteria from the Nordic score had significant predictive value.

Childhood ITP recovery score (25), has demonstrated its score accuracy from the inclusion of two continuous variables (age and level of thrombocytopenia), which could correctly predict transient vs. persistent ITP. In this score, the absence of a subtle onset is the strongest predictor for transient ITP.

Larger sample size, with longer follow up as well as a combination with genetic biomarkers may improve ITP outcome predictability in the future. This disease is the illustration of the concept of an individualized approach and the patient centered decision making. It is probably unanimously acknowledged that the therapeutic goal is to use minimum of medication for the best results.

## Author Contributions

CG, E-LS, TR, VVL, IM, and A-VI: conceptualization. CG, E-LS, AMo, VR, A-VI, and AMu: writing—original draft preparation. CG, E-LS, TR, and IM: writing—review and editing. CG: visualization and supervision. All authors have read and agreed to the published version of the manuscript.

## Conflict of Interest

The authors declare that the research was conducted in the absence of any commercial or financial relationships that could be construed as a potential conflict of interest.

## Publisher's Note

All claims expressed in this article are solely those of the authors and do not necessarily represent those of their affiliated organizations, or those of the publisher, the editors and the reviewers. Any product that may be evaluated in this article, or claim that may be made by its manufacturer, is not guaranteed or endorsed by the publisher.
