# Supplementary figures and images for: The Use of Clinical Scores in the Management of Immune Thrombocytopenic Purpura in Children
Source: Front Pediatr. 2022 May 9;10:870064. doi: 10.3389/fped.2022.870064 (PMC9125152; doi:10.3389/fped.2022.870064)

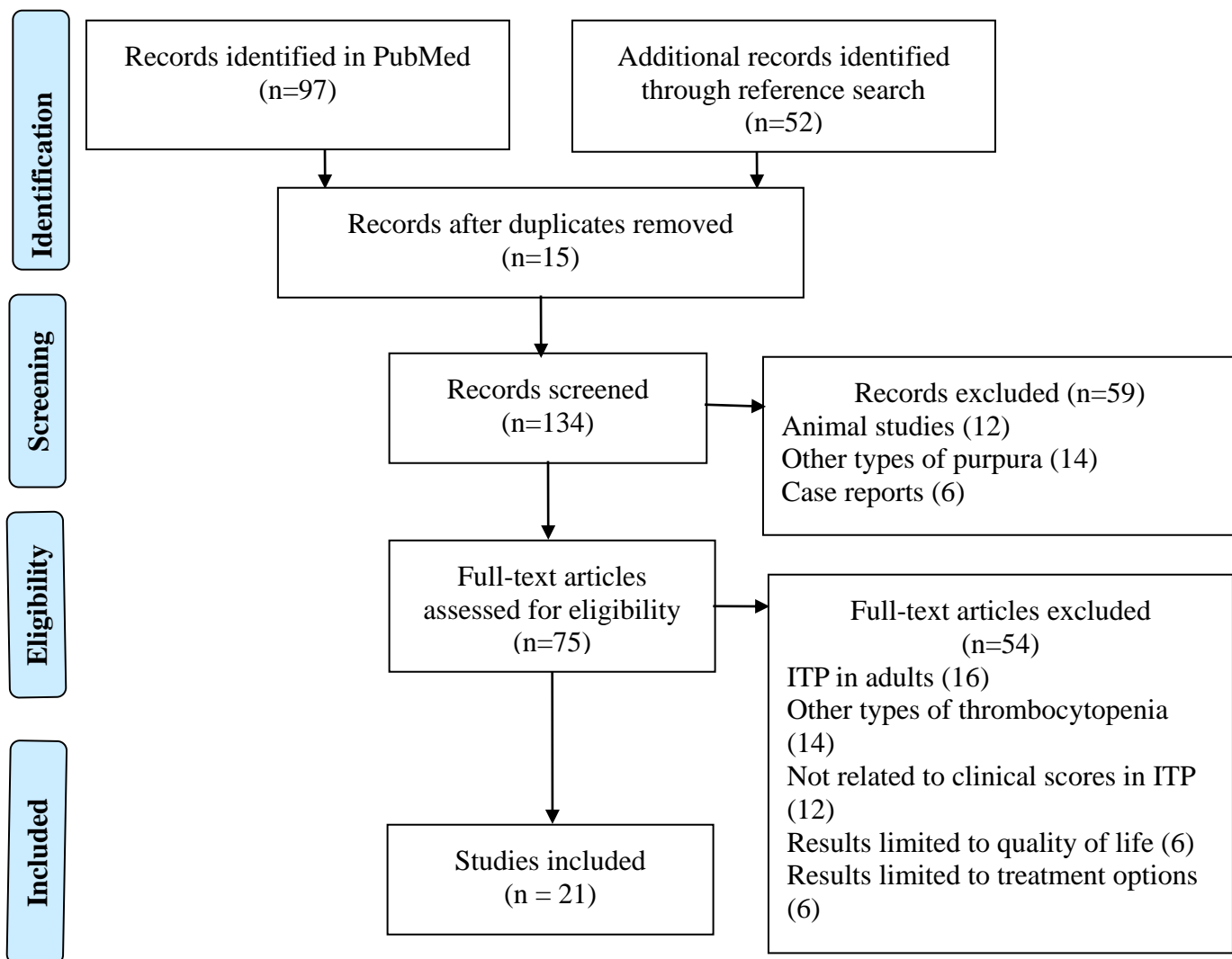

Supplement: Supplementary file 1 [file Image_1.pdf]
